# Expression of mutant alpha-synuclein modulates microglial phenotype in vitro

**DOI:** 10.1186/1742-2094-8-44

**Published:** 2011-05-09

**Authors:** Lalida Rojanathammanee, Eric J Murphy, Colin K Combs

**Affiliations:** 1 Department of Pharmacology, Physiology, & Therapeutics, University of North Dakota School of Medicine and Health Sciences, 504 Hamline Street, Neuroscience Building, Grand Forks, ND 58203, USA

## Abstract

**Background:**

Increased reactive microglia are a histological characteristic of Parkinson's disease (PD) brains, positively correlating with levels of deposited α-synuclein protein. This suggests that microglial-mediated inflammatory events may contribute to disease pathophysiology. Mutations in the gene coding for α-synuclein lead to a familial form of PD. Based upon our prior findings that α-synuclein expression regulates microglial phenotype we hypothesized that expression of mutant forms of the protein may contribute to the reactive microgliosis characteristic of PD brains.

**Methods:**

To quantify the effects of wild type and mutant α-synuclein over-expression on microglial phenotype a murine microglial cell line, BV2, was transiently transfected to express human wild type (WT), and mutant α-synuclein (A30P and A53T) proteins. Transfected cells were used to assess changes in microglia phenotype via Western blot analysis, ELISA, phagocytosis, and neurotoxicity assays.

**Results:**

As expected, over-expression of α-synuclein induced a reactive phenotype in the transfected cells. Expression of α-synuclein increased protein levels of cycloxygenase-2 (Cox-2). Transfected cells demonstrated increased secretion of the proinflammatory cytokines, tumor necrosis factor-α (TNF-α) and interleukin-6 (IL-6), as well as increased nitric oxide production. Transfected cells also had impaired phagocytic ability correlating with decreased protein levels of lysosomal-associated membrane protein 1 (LAMP-1). In spite of the increased cytokine secretion profile, the transfected cells did not exhibit increased neurotoxic ability above control non-transfected BV2 cells in neuron-microglia co-cultures.

**Conclusions:**

These data demonstrated that over-expression of α-synuclein drives microglial cells into a form of reactive phenotype characterized by elevated levels of arachidonic acid metabolizing enzymes, cytokine secretion, and reactive nitrogen species secretion all superimposed upon impaired phagocytic potential.

## Background

Microglia are a dynamic immune cell population of the central nervous system (CNS) [1-3]. They are involved in chemotaxis, phagocytosis, and proinflammatory cytokine secretion [4,5] as components of their surveillance function. A number of chronic neurodegenerative diseases, including Parkinson's disease (PD), Alzheimer's disease, and multiple sclerosis display an apparently aberrant microglial behavior that is hypothesized to contribute to disease progression [6-8]. Specifically, microglia appear to have a chronically activated phenotype exemplified by increased levels of various proinflammatory markers as well as elevated cytokine secretion.

It is interesting to note that PD brains have been characterized by progressive loss of dopaminergic neurons in the substantia nigra par compacta (SNpc) [9,10], a region with the reportedly highest density of brain microglia [11]. It is, therefore, not surprising that increased numbers of reactive microglia in the substantia nigra are characteristic of disease and reactive microglia numbers expand to other brain regions during progressive neuron loss and disease [12,13].

To explore the possibility that microglial activation plays a causative role in the proinflammatory and neurodegenerative changes observed in PD, we elected to model a familial form of disease which results from over-expression of wild type or mutant α-synuclein [14-16]. α-Synuclein is a 140 amino acid protein that is highly expressed in the central nervous system immuno-localizing to presynaptic terminals of neurons [17-19] as well as glia and macrophage [20-24]. α-Synuclein reportedly functions in regulating synaptic vesicle pools [18], interacts with a variety of proteins [25-27], and regulates lipid metabolism [28,29]. We have also demonstrated that α-synuclein expression regulates the behavior of microglia [30]. A reactive microglial phenotype was increased in α-synuclein knock-out compared to wild type microglia [30]. However, whether over-expression of wild type or mutant forms of α-synuclein may also regulate microglial phenotype remains unclear.

In order to characterize the behavior of microglia that over-express wild type or mutant α-synuclein, the mouse microglial cell line, BV2, was transiently transfected to express either human wild type (WT), A30P, or A53T mutant α-synuclein to assess the impact of intracellular over-expression on microglial behavior, rather than phenotype changes due to stimulation with extracellular α-synuclein. This study offers insight into varied mechanisms in which α-synuclein may contribute to phenotype changes in microglia during disease.

## Methods

### Materials

The anti-α synuclein antibody was obtained from Covance (Emerryville, CA). The anti-Cox-2, anti-LAMP-1, anti-actin and anti-GAPDH antibodies were purchased from Santa Cruz Biotechnology. Anti-PLD1 and PLD2 antibodies were purchased from Abcam (Cambridge, MA). The anti-Cox-1 antibody was purchased from Cayman (Ann Arbor, MI). The anti-MAP2 antibody was from Sigma (St Louis, MO). Anti-mouse, anti-rabbit and anti-goat horseradish peroxidase conjugated secondary antibodies were purchased from Santa Cruz Biotechnology (Santa Cruz, CA). Lipopolysaccharide (LPS) was purchased from Santa Cruz Biotechnology (Santa Cruz, CA). FITC-labeled Escherichia coli (K-12 strain) Bioparticles were purchased from Molecular Probes (Eugene, OR). The LDH assay kit was obtained from Promega (Madison, WI).

### Microglial Culture

The BV2 immortalized microglial cell line [31] was obtained from Dr. Gary E. Landreth (Cleveland, OH). BV2 cells were grown in Dulbecco's modified Eagle's medium:Nutrient Mixture F-12 Ham (DMEM/F-12) (Gibco RBL, Rockville, MD) supplemented with 5% horse serum (Equitech-Bio, Inc., Kerrville, TX) and 10% fetal bovine serum (U.S. Biotechnologies Inc., Parkerford, PA) and 1.5 μg/mL penicillin/streptomycin/neomycin in a humidified atmosphere of 5% CO_2 _and 95% air at 37°C.

### Transient Transfection

BV2 cells were transiently transfected with constructs (parent construct pcDNA3.1) containing cDNAs coding for human WT, A30P, or A53T α-synuclein (1 × 10^6 ^cells, 2 μg DNA per transfection) using an Amaxa Mouse Macrophage Nucleofection Kit (Lonza Group Ltd, Switzerland) according to the manufacturer's protocol. Constructs were generously provided by Dr. Nelson Cole (NIH). Transfected cells were plated at 1 × 10^6 ^cells/condition in serum containing DMEM/F12 and harvested after 48 hours post-transfection.

### Neuron Culture

Primary cortical neuron cultures were generated as previously described from cortices of embryonic day 16 C57BL/6 mice [32]. Meninges-free cortices were isolated, trypsinized and plated onto poly-L-lysine-coated (0.05 mg/mL) tissue culture wells (260 cell/mm^2^) for 7 days. The neuronal growth media was Neurobasal media supplemented with B27 and glutamine (Life Technologies, Rockville, MD, USA), which consistently provide neuronal cultures that are at least 95% pure. Culture purity was routinely evaluated by cell counting after immunostaining, to identify the neuronal cytoskeletal protein, microtubule-associated protein 2 (MAP2).

### Western Blot

To perform Western blot analyses, BV2 cells were untreated, mock transfected or transfected to express WT, A30P, or A53T α-synuclein for 48 hours. At 48 hours post-transfection cells were lysed with RIPA buffer, sonicated, and centrifuged at 14,000 RPM, 4°C for 10 minutes. Protein concentrations were quantitated using the method of Bradford [33]. Proteins were resolved by 10% or 15% SDS-PAGE and then transferred to PVDF membrane and Western blotted using anti-α-synuclein, anti-cPLA_2_, anti-Cox-1, anti-Cox-2, anti-PLD1, anti-PLD2, anti-LAMP-1, anti-actin or anti-GAPDH antibodies, followed by incubation with horseradish peroxidase-conjugated secondary antibodies. Antibody binding was visualized using enhanced chemiluminescence (Pierce, Rockford, IL). Experiments were repeated 5 independent times. To quantify protein levels, optical density (O.D.) of protein bands were normalized against their respective loading control (GAPDH or actin) using Adobe Photoshop software (Adobe Systems, San Jose, CA). Ratios were averaged for all five experiments (± SD) for statistical analysis.

### Enzyme Linked Immunosorbent Assay (ELISA)

The concentrations of secreted TNF-α and IL-6 from BV2 cultures were determined using commercially available mouse TNF-α and IL-6 colorimetric sandwich ELISA reagents purchased from R & D Systems (Minneapolis, MN). Briefly, cells were transfected and then stimulated with or without 25 ng/mL LPS (Sigma) at 48 hours post-transfection. Media was transferred to an ELISA plate and the levels of TNF-α and IL-6 were detected according to the manufacturer protocol. Experiments were performed with 8 replicates per condition and repeated three times to identify mean values (± SD).

### Griess Assay

The levels of nitrite secreted from BV2 cells were detected using Griess reagent obtained from Alexis Biochemicals (San Diego, CA). Briefly, after 48 hours post-transfection, media was transferred to 96 well plates and incubated with Griess reagent for 10 minute at room temperature. The nitrite levels were read via microplate reader at 546 nm. Experiments were performed with 8 replicates per condition and repeated three times to identify mean values (± SD).

### Phagocytosis Assay

Phagocytosis was quantified by measuring the uptake of a FITC-labeled bioparticle. Briefly, transfected BV2 cells, in 96 well plates, were incubated with or without FITC-labeled bioparticle (0.25 mg/mL) for 3 hours. To quench the signal from extracellular or outer plasma membrane associated bioparticle, medium was removed and the cells were rinsed with 0.25 mg/mL Trypan blue in phosphate buffer saline (PBS). Intracellular fluorescence was read via fluorescent plate reader (Bio-Tek, Winooski, Vermont) at 480 nm excitation and 520 nm emission. Experiments were performed with 8 replicates per condition and repeated a minimum of three times to determine mean values (±SD).

### Lactate Dehydrogenase (LDH) Assay

LDH release was measured a using CytoTox 96 non-radioactive cytotoxicity assay kit according to the manufacturer protocol (Promega). Optical densities were measured by a microplate reader at 490 nm. Each condition was performed with a replicate of 8 and mean values (± SD) from three independent experiments were determined.

### Microglial-mediated Neurotoxicity Assays

To assess the microglia-mediated neurotoxicity, neurons were co-incubated either alone or with mock transfected, WT, A30P, or A53T transfected BV2 cells for 72 hours. Neurons were plated onto 24 well plates (40,000 cells/well) and at 7 days *in vitro *were co-cultured with BV2 cells (4,000 cells/insert) that were plated onto cell culture inserts (0.4 μm Millicell, Millipore) in Neurobasal medium with or without 25 ng/ml LPS for 72 hours. After the 72 hour incubation, neurons were fixed in 4% paraformaldehyde and immunostained with antibody recognizing the neuronal cytoskeletal protein, microtubule-associated protein 2 (MAP2). A counting grid placed on the bottom of the wells was used to determine the number of viable neurons. Neurons from 4 independent fields/well from 8 wells per condition were counted. Neurons were counted as viable if they were MAP2 positive, had a visible nuclei and immunostained processes which were at least two times the length of the cell body. Mean values (± SD) from three independent experiments were determined.

### Statistical Analysis

Mean values (± SD) for each experiment were determined and values statistically different from controls were calculated using one-way ANOVA. The Newman-Keuls multiple comparisons post-test was used to determine p-values. GraphPad Prism 4 software was used for analysis (GraphPad, San Diego, CA).

## Results

### Over-expression of mutant α-synuclein increased Cox-2 levels in BV2 cells

**Figure 1:**
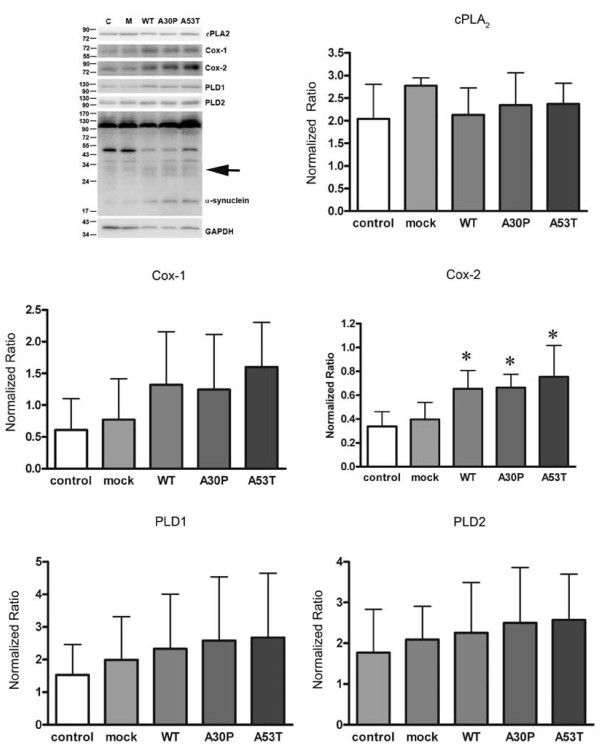
**α-synuclein transfected BV2 cells increased Cox-2 protein expression**. BV2 cells were transiently transfected to express WT, A30P, or A53T α-synuclein for 48 hours. A) Cells were lysed and Western blotted using anti-cPLA_2_, anti-Cox-1, anti-Cox-2, anti-PLD1, anti-PLD2, anti- α-synuclein, or anti-GAPDH (loading control) antibodies. Protein levels of B) cPLA_2_, C) Cox-1, D) Cox-2, E) PLD1, and F) PLD2 were quantified and normalized to GAPDH. Graphs are the average (± SD) of five independent experiments. * p < 0.05 compared to mock transfected cells.

To address whether α-synuclein over-expression modulates microglial phenotype, the murine microglial cell line, BV2, [31] was selected as an *in vitro *model of microglia because it is amenable to transient transfection for exogenous gene over-expression. BV2 cells were transfected to express human WT, or missense mutation A30P [34] or A53T [14]. Following transfection, levels of WT and mutant α-synuclein as well as several protein associated with α-synuclein function were examined via Western blot and changes due to α-synuclein over-expression were quantified. Interestingly, over-expression of α-synuclein, both wild type and mutant forms, resulted in not only the monomeric species but also an SDS-stable oligomeric form migrating between 24 and 34 kDa. Because over-expression and interaction of α-synuclein with PLD attenuates PLD activity [25,35-37] we first examined transfected cells for changes in PLD1 or PLD2 levels. Over-expression of WT and the A30P and A53T mutants had no effect on PLD1/2 protein levels suggesting that although α-synuclein expression or function may regulate PLD activities it is not involved in regulating enzyme expression or turnover in these cells (Figure 1).

In addition to a role in regulating PLD activity, a variety of studies from our group as well as others have demonstrated that α-synuclein associates with lipid membranes and its expression regulates lipid metabolism [29,38-46]. For example, α-synuclein expression modulates brain arachidonic acid metabolism and its absence produces deficiencies in arachidonic acid recycling [42] in α-synuclein knockout mouse brains relative to wild type brains resulting in elevated prostaglandin formation after an ischemic insult [39]. To examine whether over-expression of α-synuclein altered arachidonic acid metabolism in the cells, several enzymes involved in regulating arachidonic acid conversion to prostanoids were examined, cPLA_2_, Cox-1 and Cox-2. Transfected cells demonstrated no change in cPLA_2 _or Cox-1 levels, but WT, A30P, and A53T mutant α-synuclein over-expression significantly increased Cox-2 protein levels compared to mock transfected control cells (Figure 1). These data support the notion that α-synuclein over-expression can drive microglia to acquire a reactive phenotype which appears to be exacerbated by mutant protein expression and is focused upon alteration of proinflammatory prostaglandin production.

### Over-expression of α-synuclein was not toxic to BV2 cells

**Figure 2:**
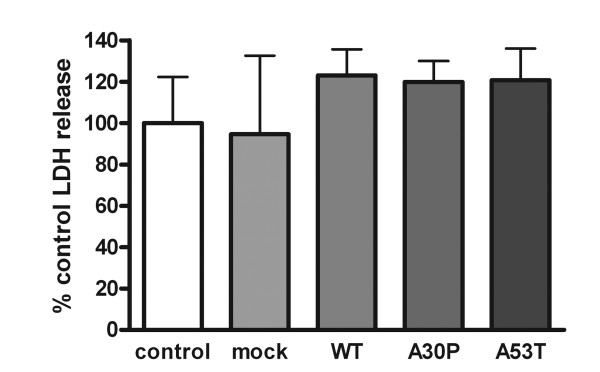
**α-synuclein over-expression was not toxic to BV2 cells**. BV2 cells were transiently transfected to express WT, A30P, or A53T α-synuclein for 48 hours. An LDH release assay was performed to determine cell viability by quantifying LDH release into the medium using commercial LDH assay reagents. Graphs are the average (± SD) of three independent experiments. Each experiment was performed with 8 replicates per condition.

Because α-synuclein over-expression can promote toxicity or increased vulnerability to stressors in some cells [47-49] it was necessary to validate that expression of the exogenous α-synuclein was not inducing cell death as a component of the acquisition of a proinflammatory phenotype in BV2 cells. This is particularly relevant for microglial cells which often include a programmed death response as a culmination of their activation pathways [50-52]. Cell viability was assessed by examining enzymatic activity of lactate dehydrogenase released into the media. Although the transfection procedure produced some expected toxicity of cells compared to non-transfected controls, expression of the exogenous human α-synuclein proteins was not toxic to the cells (Figure 2). This lack of toxicity correlated with increased detectable levels of monomeric α-synuclein as well as apparent higher molecular SDS-stable oligomers ranging from 24-34 kDa but no detectable detergent insoluble aggregates (Figure 1). This suggested that a higher molecular weight or aggregate form may be required for over-expression dependent toxicity in these cells. This data demonstrated that changes in proinflammatory Cox-2 levels were not indicative of a cell death pathway induced by mutant α-synuclein over-expression.

### Over-expression of α-synuclein attenuated the phagocytic ability of BV2 cells and decreased lysosomal protein expression

**Figure 3:**
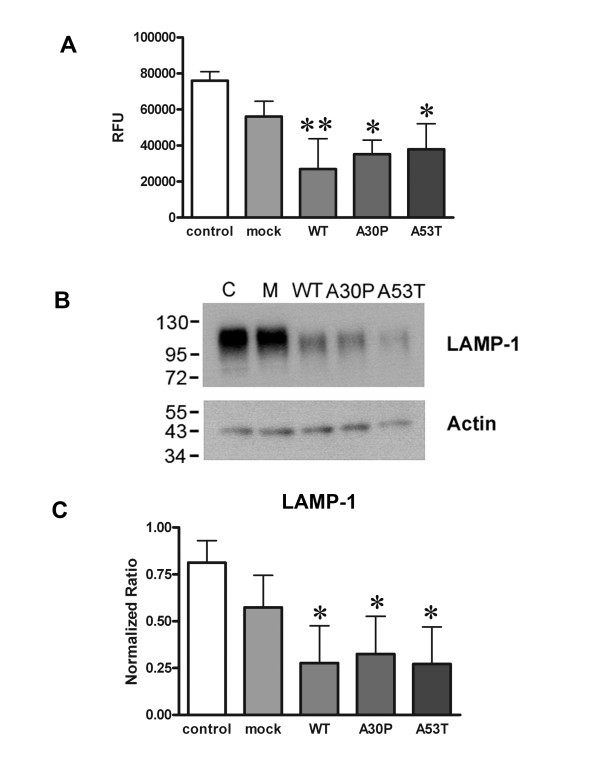
**Over-expression of α-synuclein attenuated the phagocytic ability of BV2 cells and lysosomal protein expression**. BV2 cells were transiently transfected to express WT, A30P, or A53T α-synuclein for 48 hours. A) Transfected cells were incubated with FITC-labeled E. coli bioparticles (0.25 mg/mL) for 3 hours. After incubation, the media was removed and the signal from any unphagocytosed or membrane associated particles was quenched by incubating cells with a (0.25 mg/mL) trypan blue solution for 3 minutes. The fluorescence intensity of phagocytosed particles was measured via fluorescent plate reader (RFU). Each condition was performed with 8 replicates. B) Transfected cells were also lysed and Western blotted with anti-LAMP-1 and actin (loading control) antibodies. C) Optical density of LAMP-1 immunoreactive bands were normalized against their respective actin bands and averaged (± SD) from three independent experiments. * p < 0.05 compared to mock transfected cells, **p < 0.01 compared to mock transfected cells.

Based upon several prior reports that prostaglandins can negatively regulate macrophage and microglial phagocytic ability [53-57] and our observation of increased Cox-2 protein levels in α-synuclein over-expressing cells we examined whether transfected cells would display an expected decrease in phagocytic ability. To examine changes in microglial phagocytic ability, uptake of FITC-labeled *E. coli *bioparticles was quantified from transfected cells. Consistent with the observed increase in Cox-2 protein levels, cells transfected to express WT, the A30P, or A53T mutants all displayed a significant decrease in ability to phagocytose the bioparticles compared to mock transfected cells (Figure 3A). Moreover, the decrease of phagocytic ability with over-expression correlated with a significant decrease in protein levels of the lysosomal marker protein, lysosome associated protein 1 (LAMP-1) (Figure 3B and 3C). These data again confirm that microglial over-expression of wild type or mutant α-synuclein results in an altered phenotype.

### Over-expression of α-synuclein increased proinflammatory secretion from BV2 cells

**Figure 4:**
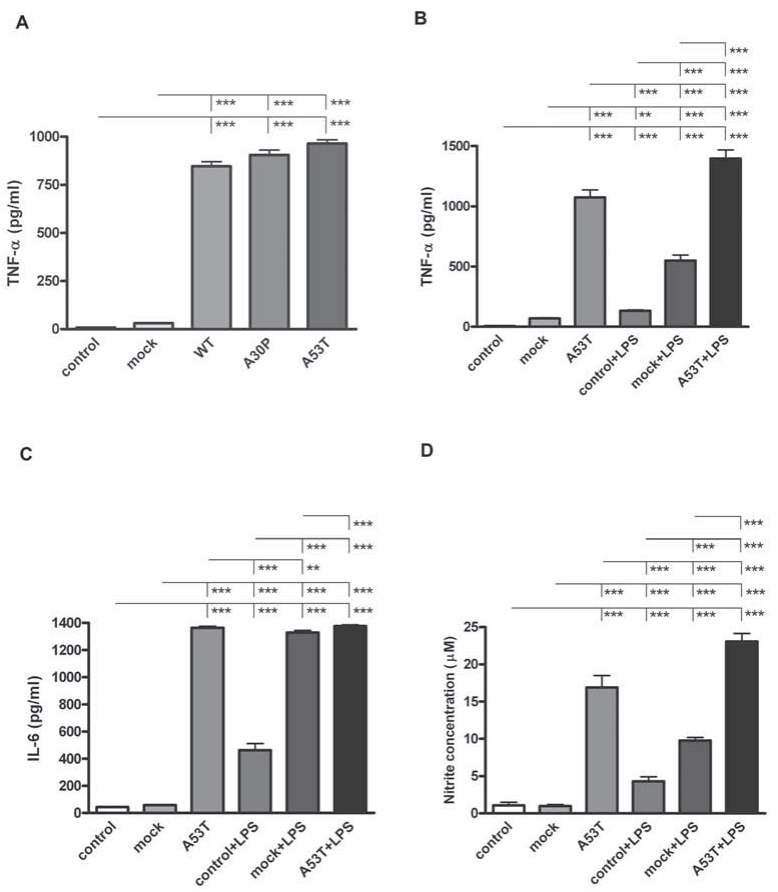
**Over-expression of α-synuclein increased TNF-α, IL-6, and nitrite levels in medium from BV2 cells**. BV2 cells were transiently transfected to express WT, A30P, or A53T alpha-synuclein for 48 hours with or without 25 ng/ml LPS stimulation. Media were collected and used for quantifying concentrations of secreted A) and B) TNF-α, C) IL-6 using a commercial mouse TNF-α and IL-6 ELISA. D) Media was also used to perform Griess assay to detect the levels of nitrite secreted from BV2 cells. Each condition was performed with 8 replicates. Graphs are the average (± SD) of three independent experiments. ** p < 0.01, *** p < 0.001.

Based upon the fact that several reports have also demonstrated that select prostaglandins can negatively regulate cytokine secretion from microglia [58-60] we next examined whether secretion of the proinflammatory cytokine, TNF-α, was altered in transfected BV2 cells. Surprisingly, levels of secreted TNF-α were significantly higher in medium from cells over-expressing both wild type and mutant α-synuclein compared to mock transfected controls (Figure 4). In order to better examine the range of secretory phenotype change due to α-synuclein over-expression, cells over-expressing the A53T mutant as a representative over-expression phenotype were stimulated with and without the proinflammatory ligand, LPS, to quantify media concentrations of not only TNF-α, but also an additional cytokine, IL-6. As expected, stimulation with LPS significantly increased secretion of both cytokines from A53T transfected cells above the levels secreted from LPS stimulated mock transfected cells (Figure 4).

In order to examine other secretory products from activated microglia we next quantified medium levels of nitrite via Griess assay as an indirect assessment of nitric oxide production by the cells. Again using A53T mutant α-synuclein over-expressing cells, we observed significantly higher levels of nitrite in the medium, both basally and with LPS stimulation above the levels observed from their respective mock-transfected counterparts (Figure 4). These data demonstrated that although Cox-2 protein levels were increased along with impaired phagocytosis, the overall secretory capacity of α-synuclein expressing microglia for TNF-α, IL-6, and nitric oxide was not compromised but instead potentiated both basally and in response to LPS stimulation. This demonstrated that the reactive phenotype of α-synuclein over-expressing cells is likely not a straight-forward consequence of elevated Cox-2 activity.

### BV2 cells over-expressing α-synuclein did not display enhanced neurotoxicity

**Figure 5:**
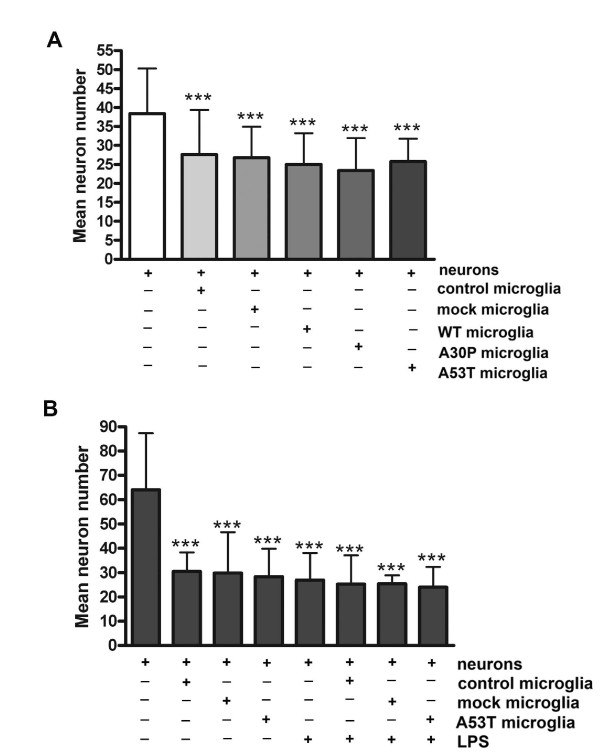
**Over-expression of α-synuclein did not increase neurotoxic secretion from BV2 cells**. BV2 cells were transiently transfected to express WT, A30P, or A53T α-synuclein then co-cultured onto a membrane insert with 7 days *in vitro *mouse cortical neurons for 72 hours in the A) absence or B) presence of 25 ng/ml LPS stimulation. After 72 hours, the inserts were removed and the neurons were fixed in 4% paraformaldehyde and immunostained with an anti-MAP2 antibody. MAP2 positive cells were counted to assess viability. Experiments were performed with 8 replicates per condition. Graphs are the average (± SD) of three independent experiments. *** p < 0.001 compared to neurons only.

Since we as well as others have demonstrated that TNF-α can alter neuronal activity and potentiate toxicity [32,61-66] it was reasonable to expect that α-synuclein over-expressing cells would demonstrate increased secretion of neurotoxic factors. To determine whether transfected BV2 cells had increased neurotoxic capacity, transfected cells were co-cultured with and without LPS in primary murine cortical neurons at 7 days *in vitro *for three days to assess effects on neuron survival. In spite of the elevated levels of cytokines and nitric oxide secreted from α-synuclein over-expressing cells, they demonstrated no increase in neurotoxic secretion above that induced by mock transfected cells with or without LPS stimulation (Figure 5). This demonstrated that although the reactive phenotype induced by α-synuclein over-expression included elevated proinflammatory secretion, this was not sufficient to induce an increased neurotoxic response *in vitro *in these particular culture conditions in which toxicity was already maximal in control BV2/neuron co-cultures.

## Discussion

This study demonstrated that over-expression of α-synuclein modulates the phenotype of a commonly used microglial model, BV2 cells. It is important to point out that these data are derived from a microglial cell line and the possibility exists that a more accurate prediction of microglial behavior in response to α-synuclein over-expression would emerge through the use of primary cells. For instance, microglia grown from transgenic mouse lines over-expressing mutant or wild type protein could be used in future work. Nevertheless, the current data set demonstrated a clear change in cellular behavior of microglial BV2 cells that involved an increase in Cox-2 protein levels in cells over-expressing human WT, mutant A30P and A53T α-synuclein. In addition, over-expression of A30P and A53T mutants as well as human WT α-synuclein decreased phagocytic ability of the BV2 cells while increasing their secretion of TNF-α, IL-6, and nitric oxide. However, in spite of these robust changes in behavior, the α-synuclein over-expressing BV2 cells did not demonstrate any increase in neurotoxic capacity.

Although extracellular α-synuclein in PD may be acting as one of the sources for the induction of microgliosis, our efforts were to identify a fundamental role for intracellular α-synuclein in regulating microglial phenotype in particularly the familial form of disease. Therefore, a distinction of our work from several prior reports is that we have examined effects of α-synuclein expression on microglial phenotype rather than effects of adding α-synuclein to microglia as a ligand. It is assumed that microgliosis occurs during disease in part due to neuronal secretion of α-synuclein which directly stimulates microglia in a fashion requiring CD36 [16,67]. Several studies demonstrate that extracellularly applied α-synuclein directly stimulates phagocytic cells such as microglia, macrophage, and monocytes to acquire a reactive phenotype characterized by a number of changes including increased production of matrix metalloproteases [68,69], increased Cox-2 levels [19], increased cytokine secretion [18], increased neurotoxin secretion [18,70], and increased cellular migration [69]. Our findings indicate that expression of α-synuclein also drives microglia into a form of activation characterized by elevated proinflammatory cytokine secretion and Cox-2 levels accompanied by impaired phagocytosis that appears unique from activation characterized by extracellular α-synuclein stimulation. These findings may help to elucidate the biology of early onset disease and indicate that microgliosis occurs as not only a reaction to neuronal death and α-synuclein secretion but may also cause neuronal dysfunction through impaired homeostasis and a proinflammatory phenotype.

Although it is our expectation that the phenotype changes observed in the BV2 cells was due to effects of α-synuclein expression we cannot exclude the possibility that a portion of the translated protein is being exocytosed into the media and acting as an extracellular ligand as others have reported [71]. It is possible that BV2 cells over-expressing α-synuclein could be secreting the protein to provide a pool for extracellular, autocrine stimulation. A prior report demonstrated that extracellular stimulation with α-synuclein compared to over-expression of α-synuclein in BV2 cells produced similar changes in cellular migration and CD44 expression [69]. This supports the idea that some component of the phenotype change we observed may be due to autocrine, feed-forward stimulation of exocytosed α-synuclein combined with the consequences of over-expression on cellular behavior. More importantly, this suggests again that microgliosis in disease is not only a consequence of α-synuclein expression by microglia but is also modified by secreted α-synuclein that could be coming from neurons but also other cells in the brain such as microglia and astrocytes [71,72]. Dissecting the differences between the two types of α-synuclein-mediated activation will offer a clearer target for attenuating the microglial contributions to disease.

Histologic data from human PD brains has demonstrated increased Cox-2 immunoreactivity within both microglia and neurons of the substantia nigra suggesting that Cox-2-dependent prostaglandin production contributes to inflammatory gliosis and neuron death [73,74]. A role for Cox-2 dependent inflammation and cell death in disease is supported by the MPTP toxin model of PD in which Cox-2 activity is required for the observed neuronal death [75-77]. Although a myriad of mechanisms are feasible, one intriguing idea is that the increased Cox-2 activity leads to dopamine oxidation and subsequent α-synuclein accumulation as Lewy bodies [78]. We now extend this data to demonstrate that α-synuclein over-expression of particularly the missense mutant forms increased microglial Cox-2 expression.

The consequences of increased Cox-2 activity in microglia are several. For instance, Zhang et al. (2005) demonstrated that microglial stimulation with extracellular aggregated α-synuclein enhances microglial production of PGE_2 _required for subsequent neurotoxin secretion [70]. On the other hand, PGE_2 _stimulation inhibits TNF-α secretion from BV2 and microglial cells [58-60] and impairs phagocytic ability [79]. Although the specific prostaglandin formation and function downstream of increased microglial Cox-2 expression or activity is far from resolved our data correlates well with an emerging theme that arachidonic acid metabolism is disrupted during disease due to a fundamental role of α-synuclein in regulating lipid metabolism [39,42]. In addition, other studies have demonstrated that a disruption in particular prostaglandin levels correlates with disease, α-synuclein expression, and in some cases induces disease phenotype [39,80-83]. Therefore, alterations in arachidonic acid metabolism appear central to both sporadic and familial disease across cell several types including microglia and neurons and defining the specific production patterns and consequences of individual prostaglandins will be critical in defining their role during PD.

Another interesting phenotype change produced by over-expression of α-synuclein was the attenuated ability of microglia to phagocytose the *E. coli *bioparticles. This correlated with a significant decrease in LAMP-1 levels suggesting lysosomal dysfunction, at least, is a component of the uptake problem. It is clear that α-synuclein has a role in modulating vesicular trafficking in other cells types [84,85] so it is not unreasonable that a similar regulatory role exists in phagocytic cells such as microglia. For example, *in vitro *studies demonstrate that microglia are capable of taking up α-synuclein, in particular its monomeric form, in what appears to be a classic-clathrin dependent mechanism [5,86]. Indeed the monomeric protein facilitates overall microglial phagocytic ability, while the aggregate form attenuates phagocytosis [5]. It is possible that α-synuclein expression by microglia actually attenuates the ability of these cells to take up aggregate α-synuclein thus contributing to disease.

Another interesting possibility is that the decrease in phagocytic ability by α-synuclein over-expressing cells is due to an increase in Cox-2 mediated prostaglandin formation. Extracellular α-synuclein aggregates stimulate microglia *in vitro *and attenuate their ability to phagocytose the aggregates in a fashion requiring PGE_2 _stimulation of its EP2 receptors [79]. In addition, PGE_2 _stimulation of its EP2 receptor downregulates microglial ability to take up another aggregate protein, beta amyloid [53,54]. This collectively supports the idea that specific prostaglandin stimulations modulate microglial behavior. Therefore, over-expression of α-synuclein may impair the general homeostatic role of microglia as brain phagocytes and while certainly of relevance to PD this implicates a broader role for this protein in how microglia function in the brain.

It is somewhat surprising that the α-synuclein over-expressing cells did not demonstrate increased neurotoxic capacity in our co-culture paradigm with and without LPS stimulation. One possibility is that altered culture conditions including different cellular ratios or incubation times as well as the use of primary microglia instead of BV2 cells may produce different results in neuronal or synaptic viability. However, the fact that cells over-expressing α-synuclein demonstrated significant changes in secretory phenotype without a correlating change in neurotoxic capacity in the conditions tested is still of importance. For example, there is certainly *in vivo *evidence that microgliosis can occur as a consequence of α-synuclein expression that involves proinflammatory change without robust neuron death. Specifically, over-expression of α-synuclein results in an early increase in microgliosis prior to neuron death in some rodent models of disease [16,67]. Another study has shown using an adeno-associated virus model to over-express α-synuclein in mice that a robust increase in microgliosis as well as T and B cell infiltration occurs in the absence of any robust neurodegeneration [87]. Others report that the reactive microglial phenotype varies in response to neuronal α-synuclein expression depending upon whether or not neurons are dying [88]. Collectively, it appears that microgliosis *in vitro *and *in vivo *may be heterogeneous in response to α-synuclein expression.

For instance microgliosis during disease may be a consequence of extracellular α-synuclein stimulation as a distinct and additional mechanism of activation than that induced by over-expression of α-synuclein. This suggests that a heterogeneous range of microgliosis phenotypes exist during disease and across brain regions that have yet to be fully described in which each type of activation may contribute differently to disease. Extracellular stimulation of microglia with exocytosed α-synuclein may be responsible for a form of gliosis while direct effects on microglial phenotype due to expression of α-synuclein may produce a similar yet unique phenotype. Future efforts examining primary microglia rather than BV2 cells over-expressing α-synuclein in neuron microglia co-culture using different cellular ratios, neuronal populations, or time points may provide a clearer picture of changes in neuron viability or, more importantly, synaptic integrity. It will be important to determine whether a temporal effect of either form of gliotic stimulation, α-synuclein over-expression or extracellular stimulation, occurs during disease and whether or not they provide combined or singular insults to the neuronal death that occurs.

## Conclusions

These data demonstrate that α-synuclein over-expression forces microglia to acquire a particular form of reactive phenotype characterized by increased cytokine and nitric oxide secretion and Cox-2 levels yet impaired phagocytic ability. This suggests that microglial activation by α-synuclein may contribute to the neuroinflammatory component of disease.

## Authors' contributions

L.R. was responsible for conducting all experiments, interpreting data, and writing the initial version of the manuscript.

E.J.M. was involved in data interpretation and revising the final version of the manuscript.

C.K.C. was involved in overall experiment design, data interpretation, and revising the final version of the manuscript.

All authors have read and approved of the final version of the manuscript.

## Competing interests

The authors declare that they have no competing interests.
